# Supplementary material for: The human gut and groundwater harbor non-photosynthetic bacteria belonging to a new candidate phylum sibling to Cyanobacteria
Source: eLife. 2013 Oct 1;2:e01102. doi: 10.7554/eLife.01102 (PMC3787301; doi:10.7554/eLife.01102)
Supplement: Figure 4—source data 1. — Analysis is based on the KEGG Module database (Kanehisa and Goto, 2000). DOI: http://dx.doi.org/10.7554/eLife.01102.011 [file elife01102s002.docx]

**Figure 4 – Source data 1.** Examination of enzymes (steps) in near-complete KEGG based modules shared among or unique to subsurface ACD20 and gut Melainabacteria genomes MEL.A1, MEL.B1, and MEL.B2. Analysis is based on the KEGG Module database ([Kanehisa and Goto 2000](#_ENREF_1)).

| ***Pathway*** | ***# Steps*** | ***ACD20*** | ***MEL.A1*** | ***MEL.B1*** | ***MEL.B2*** | ***Description*** |
| --- | --- | --- | --- | --- | --- | --- |
| ***Pathways present in both subsurface and gut MEL genomes*** | | | | | | |
| M00178 | 52 | 49 | 51 | 52 | 52 | Ribosome, bacteria |
| M00019 | 8 | 8 | 5 | 5 | 8 | Leucine biosynthesis, pyruvate => 2-oxoisovalerate => leucine |
| M00003 | 8 | 7 | 6 | 6 | 5 | Gluconeogenesis, oxaloacetate => fructose-6P |
| M00001 | 10 | 9 | 8 | 8 | 8 | Glycolysis (Embden-Meyerhof pathway), glucose => pyruvate |
| M00157 | 8 | 7 | 8 | 7 | 8 | F-type ATPase, bacteria |
| M00164 | 8 | 7 | 8 | 7 | 8 | ATP synthase |
| M00016 | 9 | 7 | 6 | 6 | 6 | Lysine biosynthesis, succinyl-DAP pathway, aspartate => lysine |
| M00096 | 8 | 6 | 7 | 7 | 7 | C5 isoprenoid biosynthesis, non-mevalonate pathway |
| M00002 | 6 | 6 | 5 | 5 | 5 | Glycolysis, core module involving three-carbon compounds |
| M00260 | 6 | 6 | 5 | 5 | 6 | DNA polymerase III complex, bacteria |
| M00239 | 5 | 4 | 5 | 4 | 4 | Peptides/nickel transport system |
| M00018 | 5 | 5 | 5 | 5 | 5 | Threonine biosynthesis, aspartate => homoserine => threonine |
| M00115 | 5 | 5 | 5 | 5 | 5 | NAD biosynthesis, aspartate => NAD |
| M00123 | 4 | 3 | 4 | 4 | 4 | Biotin biosynthesis, pimeloyl-CoA => biotin |
| M00022 | 4 | 4 | 4 | 4 | 4 | Shikimate pathway, phosphoenolpyruvate + erythrose-4P => chorismate |
| M00049 | 4 | 4 | 4 | 4 | 4 | Adenine ribonucleotide biosynthesis, IMP => ADP, ATP |
| M00311 | 4 | 4 | 4 | 4 | 4 | 2-oxoglutarate:ferredoxin oxidoreductase |
| M00063 | 3 | 3 | 3 | 3 | 2 | CMP-KDO biosynthesis |
| M00119 | 3 | 2 | 3 | 3 | 3 | Pantothenate biosynthesis, valine/L-aspartate => pantothenate |
| M00007 | 4 | 3 | 3 | 3 | 3 | Pentose phosphate pathway, non-oxidative phase, fructose 6P => ribose 5P |
| M00207 | 4 | 3 | 3 | 3 | 3 | Putative multiple sugar transport system |
| M00120 | 3 | 3 | 3 | 3 | 3 | Coenzyme A biosynthesis, pantothenate => CoA |
| M00125 | 3 | 3 | 3 | 3 | 3 | Riboflavin biosynthesis, GTP => riboflavin/FMN/FAD |
| M00210 | 3 | 3 | 3 | 3 | 3 | Putative ABC transport system |
| M00118 | 2 | 2 | 1 | 2 | 2 | Glutathione biosynthesis, glutamate => glutathione |
| M00052 | 3 | 2 | 2 | 2 | 2 | Pyrimidine ribonucleotide biosynthesis, UMP => UDP/UTP,CDP/CTP |
| M00140 | 3 | 2 | 2 | 2 | 2 | C1-unit interconversion, prokaryotes |
| M00183 | 3 | 2 | 2 | 2 | 2 | RNA polymerase, bacteria |
| M00015 | 2 | 2 | 2 | 2 | 2 | Proline biosynthesis, glutamate => proline |
| M00021 | 2 | 2 | 2 | 2 | 2 | Cysteine biosynthesis, serine => cysteine |
| M00125 | 2 | 2 | 2 | 2 | 2 | Riboflavin biosynthesis, GTP => riboflavin/FMN/FAD |
| M00256 | 2 | 2 | 2 | 2 | 2 | Cell division transport system |
| ***Pathways only present in subsurface MEL genome (ACD20)*** | | | | | | |
| M00121 | 6 | 5 | 0 | 0 | 0 | Heme biosynthesis, glutamate => protoheme/siroheme |
| M00048 | 9 | 8 | 4 | 4 | 4 | Inosine monophosphate biosynthesis, PRPP + glutamine => IMP |
| M00026 | 6 | 4 | 0 | 0 | 0 | Histidine biosynthesis, PRPP => histidine |
| M00122 | 5 | 4 | 0 | 0 | 1 | Cobalamin biosynthesis, cobinamide => cobalamin |
| M00028 | 4 | 4 | 0 | 0 | 0 | Ornithine biosynthesis, glutamate => ornithine |
| M00245 | 4 | 4 | 0 | 0 | 0 | Cobalt transport system |
| M00029 | 5 | 3 | 0 | 0 | 1 | Urea cycle |
| M00246 | 4 | 3 | 0 | 0 | 0 | Nickel transport system |
| M00023 | 3 | 3 | 0 | 0 | 0 | Tryptophan biosynthesis, chorismate => tryptophan |
| M00050 | 4 | 4 | 2 | 2 | 2 | Guanine ribonucleotide biosynthesis IMP => GDP,GTP |
| M00051 | 3 | 2 | 0 | 0 | 0 | Uridine monophosphate biosynthesis, glutamine (+ PRPP) => UMP |
| M00228 | 3 | 3 | 1 | 1 | 1 | Putative glutamine transport system |
| M00325 | 3 | 2 | 0 | 0 | 1 | alpha-Hemolysin/cyclolysin transport system |
| M00250 | 2 | 2 | 0 | 0 | 0 | Lipopolysaccharide transport system |
| M00255 | 2 | 2 | 0 | 0 | 1 | Lipoprotein-releasing system |
| ***Pathways only present in gut MEL genome(s)*** | | | | | | |
| M00248 | 2 | 0 | 0 | 2 | 1 | Putative antibiotic transport system |

1. Kanehisa M, Goto S. 2000. KEGG: kyoto encyclopedia of genes and genomes. *Nucleic Acids Res* **28**: 27-30. doi: 10.1093/nar/28.1.27.
