# Supplementary material for: The human gut and groundwater harbor non-photosynthetic bacteria belonging to a new candidate phylum sibling to Cyanobacteria
Source: eLife. 2013 Oct 1;2:e01102. doi: 10.7554/eLife.01102 (PMC3787301; doi:10.7554/eLife.01102)
Supplement: Figure 4—source data 2. — If the gene occurs in both the ACD20 and gut genomes, the reported annotation is based on ACD20. DOI: http://dx.doi.org/10.7554/eLife.01102.012 [file elife01102s003.docx]

**Figure 4 – Source data 2.** Gene annotations corresponding to the numbers in **Figure 4**. If the gene occurs in both the ACD20 and gut genomes, the reported annotation is based on ACD20.

| **Gene Number (COG by Phylogeny)** | **Metabolic Process** |
| --- | --- |
|  | **EMP Glycolysis** |
| 1 | ROK family glucokinase; K00845 [EC:2.7.1.2] |
| 2 | Glucose-6-phosphate isomerase; K01810 [EC:5.3.1.9] |
| 3 | Diphosphate-fructose-6-phosphate 1-phosphotransferase; K00895 [EC:2.7.1.90] |
| 4 | Fructose-1,6-bisphosphate aldolase, class II (EC:4.1.2.13); K01624 [EC:4.1.2.13] |
| 5 | Triosephosphate isomerase; K01803 [EC:5.3.1.1] |
| 6 | Glyceraldehyde-3-phosphate dehydrogenase, type I; K00134 [EC:1.2.1.12] |
| 7 | Phosphoglycerate kinase (EC:2.7.2.3); K00927 [EC:2.7.2.3] |
| 8 | Phosphoglycerate mutase, 2,3-bisphosphoglycerate-independent; K15633 [EC:5.4.2.1] |
| 9 | Enolase (2-phosphoglycerate dehydratase); K01689 [EC:4.2.1.11] |
| 10 | Pyruvate kinase; K00873 [EC:2.7.1.40] |
| 11 | Pyruvate dehydrogenase E1; EC:1.2.4.1] |
| 12 | Pyruvate dehydrogenase E2; [EC:2.3.1.12] |
| 13 | Pyruvate dehydrogenase E3; [EC:1.8.1.4] |
| 14 | Formate acetyltransferase; K00656 [EC:2.3.1.54] |
|  | **Fermentation end-products: Carbon** |
| 15 | acdAB; acetyl-CoA synthetase (ADP-forming), alpha and beta subunit fusion (EC:6.2.1.13) |
| 16 | Acetaldehyde dehydrogenase ; K04072 |
| 17 | Alcohol dehydrogenase; K13953 alcohol dehydrogenase, propanol-preferring [EC:1.1.1.1] |
| 18 | Acetyl-CoA acetyltransferase or thiolase; [EC 2.3.1.16]; K00626 |
| 19 | 3-hydroxybutyrate dehydrogenase; K00019 3-hydroxybutyrate dehydrogenase [EC:1.1.1.30] |
| 20 | Enoyl-CoA hydratase/isomerase; K01692 enoyl-CoA hydratase [EC:4.2.1.17] |
| 21 | Acyl-CoA dehydrogenase domain protein; K00248 butyryl-CoA dehydrogenase [EC:1.3.99.2] |
| 22 | Lactate dehydrogenase |
|  | **TCA and rTCA cycle carbon interconversions** |
| 23 | Citrate synthase; [EC:2.3.3.8 or 2.3.3.1] |
| 24 | Aconitate hydratase; [EC:4.2.1.3] |
| 25 | Isocitrate dehydrogenase NAD-dependent; K00030 [EC:1.1.1.41] |
| 26 | 2-oxoglutarate dehydrogenase |
| 27 | Succinyl-CoA ligase |
| 28 | Succinate dehydrogenase; [EC:1.3.99.1] |
| 29 | Succinate dehydrogenase; [EC:1.3.99.1] |
| 30 | Succinate dehydrogenase; [EC:1.3.99.1] |
| 31 | Fumarate hydratase; K01676 [EC:4.2.1.2] |
| 32 | Malate dehydrogenase; [EC:1.1.1.37] |
| 33 | 2-oxoglutarate:ferredoxin oxidoreductase, subunit 1; K00174 [EC:1.2.7.3] |
| 34 | 2-oxoglutarate:ferredoxin oxidoreductase, subunit 1; K00174 [EC:1.2.7.3] |
| 35 | 2-oxoglutarate:ferredoxin oxidoreductase, subunit 1; K00174 [EC:1.2.7.3] |
| 36 | 2-oxoglutarate:ferredoxin oxidoreductase, subunit 1; K00174 [EC:1.2.7.3] |
| 37 | Citryl-CoA lyase; K01644 [EC:4.1.3.34] |
| 38 | Aspartate ammonia-lyase; K01744 aspartate ammonia-lyase [EC:4.3.1.1] |
| 39 | Malic enzyme (oxaloacetate-decarboxylating); K00027 [EC:1.1.1.38] |
|  | **Fermentation end-products: Hydrogen Production** |
| 40 | Electron transfer flavoprotein alpha subunit; K03522 |
| 41 | Electron transfer flavoprotein beta subunit |
| 42 | Ferredoxin Fe-only hydrogenase; [EC:1.12.7.2] |
| 43 | Iron only NADH binding motif (trimeric hydrogenase) |
| 44 | Iron-only Ferredoxin binding motif (trimeric hydrogenase) |
| 45 | Iron-only hydrogenase catalytic subunit (trimeric hydrogenase); K0033 [EC:1.6.5.3] |
| 46 | Putative NiFe Hydrogenase Subunit 1 HydA/Nqo6-like |
| 47 | Putative NiFe Hydrogenase Subunit 2 HydB/Nqo4-like |
| 48 | Putative NiFe Hydrogenase Subunit 3 hydrogenase-4 catalytic subunit; K12141 |
| 49 | Putative NiFe Hydrogenase Subunit 4 NAD-dependent dehydrogenase subunit |
| 50 | Putative NiFe Hydrogenase Subunit 5 NAD-dependent dehydrogenase subunit |
| 51 | Putative NiFe Hydrogenase Subunit 6 hydrogenase-4 component B; K12137 |
|  | **Nitrogenase and nitrogen assimilation** |
| 52 | Nitrogenase associated protein, putative NifK |
| 53 | Nitrogenase molybdenum-iron protein alpha chain NifD [EC:1.18.6.1] |
| 54 | Nitrogenase iron protein NifH; K02588 [EC:1.18.6.1] |
| 55 | Glutamine synthetase; K01915 [EC:6.3.1.2] |
| 56 | Ferredoxin-dependent glutamate synthase subunit alpha; K00265 [EC:1.4.7.1] |
| 57 | Ferredoxin-dependent glutamate synthase subunit beta; [EC:1.4.7.1] |
| 58 | Glutamate dehydrogenase (NADP+); K00262 [EC:1.4.1.4] |
|  | **Membrane Energization and Oxidative Phosphorylation** |
| 59 | 11 subunit NADH dehydrogenase P module, putative NuoA |
| 60 | 11 subunit NADH dehydrogenase P module, putative NuoH |
| 61 | 11 subunit NADH dehydrogenase P module, putative NuoJ |
| 62 | 11 subunit NADH dehydrogenase P module, putative NuoK |
| 63 | 11 subunit NADH dehydrogenase P module, putative NuoL |
| 64 | 11 subunit NADH dehydrogenase P module, putative NuoM |
| 65 | 11 subunit NADH dehydrogenase P module, putative NuoN |
| 66 | 11 subunit NAD(P)H or NADH dehydrogenase Q module, NdhH (~NuoD) |
| 67 | 11 subunit NAD(P)H or NADH dehydrogenase Q module, NdhK (~NuoB) |
| 68 | 11 subunit NAD(P)H or NADH dehydrogenase Q module, NdhJ (~NuoC) |
| 69 | 11 subunit NADH dehydrogenase Q module, NuoI (~NdhI) |
| 70 | Ferredoxin-NADP(+) reductase; K00528 [EC:1.18.1.2] |
| 71 | Na+-transporting oxaloacetate decarboxylase, beta subunit |
| 72 | Na+-transporting oxaloacetate decarboxylase, gamma subunit |
| 73 | Na+-transporting oxaloacetate decarboxylase, alpha subunit |
| 74 | Sodium/hydrogen exchanger, monovalent cation:H+ antiporter-2, CPA2 family; K03455 |
| 75 | hppA; membrane-bound proton-translocating pyrophosphatase; K01507 [EC:3.6.1.1] |
| 76 | F ATP synthase, H+-transporting ATPase; K02115 [EC:3.6.3.14] |
| 77 | F ATP synthase, H+-transporting ATPase; K02115 [EC:3.6.3.14] |
| 78 | F ATP synthase, H+-transporting ATPase; K02115 [EC:3.6.3.14] |
| 79 | F ATP synthase, H+-transporting ATPase; K02115 [EC:3.6.3.14] |
| 80 | F ATP synthase, H+-transporting ATPase; K02115 [EC:3.6.3.14] |
| 81 | F ATP synthase, H+-transporting ATPase; K02115 [EC:3.6.3.14] |
| 82 | F ATP synthase, H+-transporting ATPase; K02115 [EC:3.6.3.14] |
| 83 | F ATP synthase, H+-transporting ATPase; K02115 [EC:3.6.3.14] |
|  | **Carbon degradation and conversions** |
| 84 | Glycoside hydrolase family protein, beta-mannosidase; K01192 [EC:3.2.1.25] |
| 85 | Alpha-amylase or amylopullulanase |
| 86 | Alpha-amylase, predicted extracellular |
| 87 | Maltose alpha-D-glucosyltransferase or Trehalose/maltose hydrolase; [EC:5.4.99.16] |
| 88 | 4-alpha-glucanotransferase; [EC:2.4.1.25] |
| 89 | Glycogen/starch synthase; K00703 [EC:2.4.1.21] |
| 90 | L-iditol 2-dehydrogenase; [EC:1.1.1.14] |
| 91 | Fructokinase; [EC:2.7.1.4] |
| 92 | Transketolase; [EC:2.2.1.1] K00615 |
| 93 | Ribulose-phosphate 3-epimerase; [EC:5.1.3.1] K01783 |
| 94 | Ribose 5-phsophate isomerase A; [EC:5.3.1.6] K01808 |
| 95 | Ribokinase; [EC:2.7.1.15] K00852 |
|  | **Membrane-Localized Transporters** |
| 96 | Sodium:dicarboxylate symporter |
| 97 | Amino acid transporter/permease |
| 98 | Ammonium transporter, Amt family; K03320 |
| 99 | L-arabinose transport system permease AraQ; |
| 100 | lacG; lactose transport system permease protein LacG; K02026 |
| 101 | Multiple sugar transport system permease protein; K02025 |
| 102 | Ferric uptake regulator, Fur family; K03711 |
| 103 | Cobalt/nickel transport system permease protein |
| 104 | Arsenate reductase, ACR2; [EC:1.20.4.1] K03471 |
| 105 | Arsenite transporter, ACR3 family; K03325 |
| 106 | Heavy metal translocating P-type ATPase; [EC:3.6.3.4] K01533 |
| 107 | Cd2+/Zn2+-exporting ATPase P-type ATPase; K01534 |
