# Supplementary material for: The human gut and groundwater harbor non-photosynthetic bacteria belonging to a new candidate phylum sibling to Cyanobacteria
Source: eLife. 2013 Oct 1;2:e01102. doi: 10.7554/eLife.01102 (PMC3787301; doi:10.7554/eLife.01102)
Supplement: Figure 9—source data 1. — DOI: http://dx.doi.org/10.7554/eLife.01102.020 [file elife01102s005.docx]

**Figure 9 – Source data**. 16S rRNA gene sequence datasets used to analyze the sources of Melainabacteria.

| QIIME ID | Title in QIIME database | Region | Cyano Seqs | Total Seqs | Ref |
| --- | --- | --- | --- | --- | --- |
| **Host associated** | | | | | |
| 317 | The influence of sex handedness and washing on the diversity of hand surface bacteria S1_V183 | NA | 1 | 460244 | [[1](#_ENREF_1)] |
| 77 | A core gut microbiome in obese and lean twins | V2 | 81 | 1039357 | [[2](#_ENREF_2)] |
| 314 | Characterization of airborne microbial communities at a high elevation site and their potential to act as atmospheric ice nuclei | V2 | 0 | 4786 | [[3](#_ENREF_3)] |
| 316 | Bacterial communities of disease vectors sampled across time space and species | V2 | 10 | 177977 | [[4](#_ENREF_4)] |
| 450 | Bacterial Community Variation in Human Body Habitats Across Space and Time | V2 | 4 | 352051 | [[5](#_ENREF_5)] |
| 449 |  | V2 | 589 | 792256 |  |
| 460 | Comparison of T5KO and wildtype mice | V2 | 13 | 18251 | [[6](#_ENREF_6)] |
| 393 | Forensic identification using skin bacterial communities | V2 | 0 | 17615 | [[7](#_ENREF_7)] |
| 232 |  | V2 | 0 | 119805 |  |
| 459 | The effect of diet on the human gut microbiome: a metagenomic analysis in humanized gnotobiotic mice. | V2 | 0 | 20415 | [[8](#_ENREF_8)] |
| 454 |  | V2 | 1 | 33530 |  |
| 458 |  | NA | 0 | 93080 |  |
| 457 |  | V2 | 0 | 156891 |  |
| 455 |  | V2 | 0 | 286779 |  |
| 456 |  | V2 | 0 | 352772 |  |
| 453 |  | V2 | 76 | 416083 |  |
| 452 |  | V2 | 0 | 484260 |  |
| 451 | Crawford mice | NA | 34 | 144845 | [[9](#_ENREF_9)] |
| 391 | Burmese python gut microbiome | V2 | 2 | 319182 | [[10](#_ENREF_10)] |
| 721 | Global patterns of 16S rRNA diversity at a depth of millions of sequences per sample | V4 | 162851 | 22302948 | [[11](#_ENREF_11)] |
| 722 |  | V4 | 0 | 36395009 |  |
| 509 | Vaginal microbiome of reproductive age women | V13 | 0 | 823722 | [[12](#_ENREF_12)] |
| 395 | Delivery mode effects on newborn microbiota | V2 | 54 | 156602 | [[13](#_ENREF_13)] |
| 486 | Composition, variability, and temporal stability of the intestinal microbiota of the elderly. | V4 | 0 | 2637162 | [[14](#_ENREF_14)] |
| 101 | Succession of microbial consortia in the developing infant gut microbiome | V2 | 0 | 313172 | [[15](#_ENREF_15)] |
| 496 | Impact of diet in shaping gut microbiota revealed by a comparative study in children from Europe and rural Africa | V56 | 0 | 7596 | [[16](#_ENREF_16)] |
| 494 | Incomplete recovery and individualized responses of the human distal gut microbiota to repeated antibiotic perturbation | V13 | 10758 | 2445577 | [[17](#_ENREF_17)] |
| 349 | Human oral, gut, and plaque microbiota in patients with atherosclerosis | V2 | 50 | 381870 | [[18](#_ENREF_18)] |
| 495 | Resistant Starches Types 2 and 4 Have Differential Effects on the Composition of the Fecal Microbiota in Human Subjects | V13 | 29 | 929198 | [[19](#_ENREF_19)] |
| 929 | Bacterial communities associated with the lichen symbiosis | V4 | 0 | 16743 | [[20](#_ENREF_20)] |
| 524 | Disordered Microbial Communities in the Upper Respiratory Tract of Cigarette Smokers | V2 | 11 | 729003 | [[21](#_ENREF_21)] |
| 966 | Characterization of Bacteria in Biopsies of Colon and Stools by High Throughput Sequencing of the V2 Region of Bacterial 16S rRNA Gene in Human | V2 | 3 | 78190 | [[22](#_ENREF_22)] |
| 625 | Diet drives convergence in gut microbiome functions across mammalian phylogeny and within humans | V2 | 33 | 69097 | [[23](#_ENREF_23)] |
| 626 |  | V2 | 163 | 160692 |  |
| 550 | Moving pictures of the human microbiome | V4 | 0 | 68666081 | [[24](#_ENREF_24)] |
| 1010 | Linking Long-Term Dietary Patterns with Gut Microbial Enterotypes | V2 | 709 | 352652 | [[25](#_ENREF_25)] |
| 742 | The antibacterial lectin RegIII promotes the spatial segregation of microbiota and host in the intestine | V13 | 11 | 104551 | [[26](#_ENREF_26)] |
| 1335 | restroom surface metagenome | V2 | 54 | 378334 | [[27](#_ENREF_27)] |
| 1345 | Architectural design influences the diversity and structure of the built environment microbiome | V2 | 0 | 65571 | [[28](#_ENREF_28)] |
| 850 | Human gut microbiome viewed across age and geography |  | 0 | 1092569420 | [[29](#_ENREF_29)] |
| 967 | Improved detection of bifidobacteria with optimised 16S rRNA gene based pyrosequencing | V35 | 0 | 37977 | [[30](#_ENREF_30)] |
| 1192 | Zebrafish gut metagenome | V3 | 0 | 92463 | [[31](#_ENREF_31)] |
| 802 | MyD88-dependent signaling modulates virus susceptibility and gut microbial ecology | V2 | 620 | 339416 | [[32](#_ENREF_32)] |
| 909 | Progression of NAFLD is driven by inflammasome-mediated dysbiosis | V2 | 192 | 369519 | [[33](#_ENREF_33)] |
| 1011 | Linking Long-Term Dietary Patterns with Gut Microbial Enterotypes | V2 | 4203 | 898799 | [[25](#_ENREF_25)] |
| 867 | Finland | V2 | 182 | 929870 | [[34](#_ENREF_34)] |
| NA | UKTwins | V2 | 1138 | 2685624 | NA |
| 968 | HMP production phase 1 | V13 | 287 | 23724484 | [[35](#_ENREF_35)] |
| 969 | HMP production phase 1 | V35 | 2800 | 36784860 |  |
| 933 | Latitudinal surveys of algal-associated microorganisms | V4 | 0 | 70989436 | NA |
| **Environment** | | | | | |
| 103 | Pyrosequencing-based assessment of soil pH as a predictor of soil bacterial community structure at the continental scale | V2 | 0 | 139612 | [[36](#_ENREF_36)] |
| 104 | Soil bacterial diversity in the Arctic is not fundamentally different from that found in other biomes | V2 | 0 | 94065 | [[37](#_ENREF_37)] |
| 130 | Bacterial community structure across groundwater arsenic gradients | V2 | 1 | 31500 | [[38](#_ENREF_38)] |
| 213 | Shifts in bacterial community structure associated with inputs of low molecular weight carbon compounds to soil | V2 | 0 | 62742 |  |
| 397 | Plot-scale manipulations of organic matter inputs to soils correlate with shifts in microbial community composition in a lowland tropical rainforest | V2 | 5 | 37836 | [[39](#_ENREF_39)] |
| 619 | NEON: Directions and resources for long-term monitoring in soil microbial ecology | V4 | 87 | 372622 | NA |
| 721 | Global patterns of 16S rRNA diversity at a depth of millions of sequences per sample | V4 | 531 | 13854938 | [[40](#_ENREF_40)] |
| 816 | The effects of soil bacterial community structure on decomposition in a tropical rain forest | V2 | 0 | 1396 | [[41](#_ENREF_41)] |
| 928 | Examining the global dominant archaeal populations in soil | V4 | 15 | 14402 | [[42](#_ENREF_42)] |
| 1005 | Uncovering the mechanistic basis for soil microbial community response to altered precipitation patterns | V6 | 125 | 177838 | NA |
| 1038 | Oregon Transect | V4 | 0 | 3869679 | NA |
| 1031 | Alder/Fir | V4 | 0 | 1863345 | NA |
| 1039 | Jurelevicius Rio de Janeiro Coastline | V4 | 0 | 6377665 | NA |
| 1037 | LTSP 16S | V4 | 0 | 4464143 | NA |
| 1197, 1046, 1198 | Jansson Gulf Oil Spill Sediments | V4 | 0 | 76309188 | NA |

1. Fierer N, Hamady M, Lauber CL, Knight R (2008) The influence of sex, handedness, and washing on the diversity of hand surface bacteria. Proceedings of the National Academy of Sciences of the United States of America 105: 17994-17999. 10.1073/pnas.0807920105.

2. Turnbaugh PJ, Hamady M, Yatsunenko T, Cantarel BL, Duncan A, et al. (2009) A core gut microbiome in obese and lean twins. Nature 457: 480-484. 10.1038/nature07540.

3. Bowers RM, Lauber CL, Wiedinmyer C, Hamady M, Hallar AG, et al. (2009) Characterization of airborne microbial communities at a high-elevation site and their potential to act as atmospheric ice nuclei. Appl Environ Microbiol 75: 5121-5130. 10.1128/AEM.00447-09.

4. Jones RT, Knight R, Martin AP (2010) Bacterial communities of disease vectors sampled across time, space, and species. Isme J 4: 223-231. 10.1038/ismej.2009.111.

5. Costello EK, Lauber CL, Hamady M, Fierer N, Gordon JI, et al. (2009) Bacterial community variation in human body habitats across space and time. Science 326: 1694-1697. 10.1126/science.1177486.

6. Vijay-Kumar M, Aitken JD, Carvalho FA, Cullender TC, Mwangi S, et al. (2010) Metabolic syndrome and altered gut microbiota in mice lacking Toll-like receptor 5. Science 328: 228-231. 10.1126/science.1179721.

7. Fierer N, Lauber CL, Zhou N, McDonald D, Costello EK, et al. (2010) Forensic identification using skin bacterial communities. Proc Natl Acad Sci U S A 107: 6477-6481. 10.1073/pnas.1000162107.

8. Turnbaugh PJ, Ridaura VK, Faith JJ, Rey FE, Knight R, et al. (2009) The effect of diet on the human gut microbiome: a metagenomic analysis in humanized gnotobiotic mice. Sci Transl Med 1: 6ra14. 10.1126/scitranslmed.3000322.

9. Caporaso JG, Kuczynski J, Stombaugh J, Bittinger K, Bushman FD, et al. (2010) QIIME allows analysis of high-throughput community sequencing data. Nat Methods 7: 335-336. 10.1038/nmeth.f.303.

10. Costello EK, Gordon JI, Secor SM, Knight R (2010) Postprandial remodeling of the gut microbiota in Burmese pythons. Isme J 4: 1375-1385. 10.1038/ismej.2010.71.

11. Caporaso JG, Lauber CL, Walters WA, Berg-Lyons D, Lozupone CA, et al. (2011) Global patterns of 16S rRNA diversity at a depth of millions of sequences per sample. Proc Natl Acad Sci U S A 108 Suppl 1: 4516-4522. 10.1073/pnas.1000080107.

12. Ravel J, Gajer P, Abdo Z, Schneider GM, Koenig SS, et al. (2011) Vaginal microbiome of reproductive-age women. Proc Natl Acad Sci U S A 108 Suppl 1: 4680-4687. 10.1073/pnas.1002611107.

13. Dominguez-Bello MG, Costello EK, Contreras M, Magris M, Hidalgo G, et al. (2010) Delivery mode shapes the acquisition and structure of the initial microbiota across multiple body habitats in newborns. Proc Natl Acad Sci U S A 107: 11971-11975. 10.1073/pnas.1002601107.

14. Claesson MJ, Cusack S, O'Sullivan O, Greene-Diniz R, de Weerd H, et al. (2011) Composition, variability, and temporal stability of the intestinal microbiota of the elderly. Proc Natl Acad Sci U S A 108 Suppl 1: 4586-4591. 10.1073/pnas.1000097107.

15. Koenig JE, Spor A, Scalfone N, Fricker AD, Stombaugh J, et al. (2011) Succession of microbial consortia in the developing infant gut microbiome. Proc Natl Acad Sci U S A 108 Suppl 1: 4578-4585. 10.1073/pnas.1000081107.

16. De Filippo C, Cavalieri D, Di Paola M, Ramazzotti M, Poullet JB, et al. (2010) Impact of diet in shaping gut microbiota revealed by a comparative study in children from Europe and rural Africa. Proc Natl Acad Sci U S A 107: 14691-14696. 10.1073/pnas.1005963107.

17. Dethlefsen L, Relman DA (2011) Incomplete recovery and individualized responses of the human distal gut microbiota to repeated antibiotic perturbation. Proc Natl Acad Sci U S A 108 Suppl 1: 4554-4561. 10.1073/pnas.1000087107.

18. Koren O, Spor A, Felin J, Fak F, Stombaugh J, et al. (2011) Human oral, gut, and plaque microbiota in patients with atherosclerosis. Proc Natl Acad Sci U S A 108 Suppl 1: 4592-4598. 10.1073/pnas.1011383107.

19. Martinez I, Kim J, Duffy PR, Schlegel VL, Walter J (2010) Resistant starches types 2 and 4 have differential effects on the composition of the fecal microbiota in human subjects. PLoS One 5: e15046. 10.1371/journal.pone.0015046.

20. Bates ST, Cropsey GW, Caporaso JG, Knight R, Fierer N (2011) Bacterial communities associated with the lichen symbiosis. Appl Environ Microbiol 77: 1309-1314. 10.1128/AEM.02257-10.

21. Charlson ES, Chen J, Custers-Allen R, Bittinger K, Li H, et al. (2010) Disordered microbial communities in the upper respiratory tract of cigarette smokers. PLoS One 5: e15216. 10.1371/journal.pone.0015216.

22. Momozawa Y, Deffontaine V, Louis E, Medrano JF (2011) Characterization of bacteria in biopsies of colon and stools by high throughput sequencing of the V2 region of bacterial 16S rRNA gene in human. PLoS One 6: e16952. 10.1371/journal.pone.0016952.

23. Muegge BD, Kuczynski J, Knights D, Clemente JC, Gonzalez A, et al. (2011) Diet drives convergence in gut microbiome functions across mammalian phylogeny and within humans. Science 332: 970-974. 10.1126/science.1198719.

24. Caporaso JG, Lauber CL, Costello EK, Berg-Lyons D, Gonzalez A, et al. (2011) Moving pictures of the human microbiome. Genome Biol 12: R50. 10.1186/gb-2011-12-5-r50.

25. Wu GD, Chen J, Hoffmann C, Bittinger K, Chen YY, et al. (2011) Linking long-term dietary patterns with gut microbial enterotypes. Science 334: 105-108. 10.1126/science.1208344.

26. Vaishnava S, Yamamoto M, Severson KM, Ruhn KA, Yu X, et al. (2011) The antibacterial lectin RegIIIgamma promotes the spatial segregation of microbiota and host in the intestine. Science 334: 255-258. 10.1126/science.1209791.

27. Flores GE, Bates ST, Knights D, Lauber CL, Stombaugh J, et al. (2011) Microbial biogeography of public restroom surfaces. PLoS One 6: e28132. 10.1371/journal.pone.0028132.

28. Kembel SW, Jones E, Kline J, Northcutt D, Stenson J, et al. (2012) Architectural design influences the diversity and structure of the built environment microbiome. Isme J: 10.1038/ismej.2011.211.

29. Yatsunenko T, Rey FE, Manary MJ, Trehan I, Dominguez-Bello MG, et al. (2012) Human gut microbiome viewed across age and geography. Nature 486: 222-227. 10.1038/nature11053.

30. Sim K, Cox MJ, Wopereis H, Martin R, Knol J, et al. (2012) Improved detection of bifidobacteria with optimised 16S rRNA-gene based pyrosequencing. PLoS One 7: e32543. 10.1371/journal.pone.0032543.

31. Rawls JF, Mahowald MA, Ley RE, Gordon JI (2006) Reciprocal gut microbiota transplants from zebrafish and mice to germ-free recipients reveal host habitat selection. Cell 127: 423-433. 10.1016/j.cell.2006.08.043.

32. Larsson E, Tremaroli V, Lee YS, Koren O, Nookaew I, et al. (2012) Analysis of gut microbial regulation of host gene expression along the length of the gut and regulation of gut microbial ecology through MyD88. Gut 61: 1124-1131. 10.1136/gutjnl-2011-301104.

33. Henao-Mejia J, Elinav E, Jin C, Hao L, Mehal WZ, et al. (2012) Inflammasome-mediated dysbiosis regulates progression of NAFLD and obesity. Nature 482: 179-185. 10.1038/nature10809.

34. Koren O, Goodrich JK, Cullender TC, Spor A, Laitinen K, et al. (2012) Host Remodeling of the Gut Microbiome and Metabolic Changes during Pregnancy. Cell 150: 470-480. 10.1016/j.cell.2012.07.008.

35. HMP (2012) Structure, function and diversity of the healthy human microbiome. Nature 486: 207-214. 10.1038/nature11234.

36. Lauber CL, Hamady M, Knight R, Fierer N (2009) Pyrosequencing-based assessment of soil pH as a predictor of soil bacterial community structure at the continental scale. Applied and environmental microbiology 75: 5111-5120. 10.1128/AEM.00335-09.

37. Chu H, Fierer N, Lauber CL, Caporaso JG, Knight R, et al. (2010) Soil bacterial diversity in the Arctic is not fundamentally different from that found in other biomes. Environmental microbiology 12: 2998-3006. 10.1111/j.1462-2920.2010.02277.x.

38. Mladenov N, Zheng Y, Miller MP, Nemergut DR, Legg T, et al. (2010) Dissolved organic matter sources and consequences for iron and arsenic mobilization in Bangladesh aquifers. Environmental science & technology 44: 123-128. 10.1021/es901472g.

39. Nemergut DR, Cleveland CC, Wieder WR, Washenberger CL, Townsend AR (2010) Plot-scale manipulations of organic matter inputs to soils correlate with shifts in microbial community composition in a lowland tropical rain forest. Soil Biology and Biochemistry 42: 2153-2160. 10.1016/j.soilbio.2010.08.011.

40. Caporaso JG, Lauber CL, Walters WA, Berg-Lyons D, Lozupone CA, et al. (2011) Global patterns of 16S rRNA diversity at a depth of millions of sequences per sample. Proceedings of the National Academy of Sciences of the United States of America 108 Suppl 1: 4516-4522. 10.1073/pnas.1000080107.

41. Leff JW, Nemergut DR, Grandy AS, O'Neill SP, Wickings K, et al. (2012) The Effects of Soil Bacterial Community Structure on Decomposition in a Tropical Rain Forest. Ecosystems 15: 284-298. Doi 10.1007/S10021-011-9510-2.

42. Bates ST, Berg-Lyons D, Caporaso JG, Walters WA, Knight R, et al. (2011) Examining the global distribution of dominant archaeal populations in soil. The ISME journal 5: 908-917. 10.1038/ismej.2010.171.
